# Condom availability in high risk places and condom use: a study at district level in Kenya, Tanzania and Zambia

**DOI:** 10.1186/1471-2458-12-1030

**Published:** 2012-11-26

**Authors:** Ingvild Fossgard Sandøy, Astrid Blystad, Elizabeth H Shayo, Emmanuel Makundi, Charles Michelo, Joseph Zulu, Jens Byskov

**Affiliations:** 1 Centre for International Health, University of Bergen, Bergen, Norway; 2 Department of Public Health, University of Bergen, Bergen, Norway; 3 National Institute for Medical Research (NIMR), Dar Es Salaam, Tanzania; 4 Department of Public Health, School of Medicine, University of Zambia, Lusaka, Zambia; 5 DBL - Centre for Health Research and Development, Faculty of Life Sciences, University of Copenhagen, Frederiksberg, Denmark

**Keywords:** HIV prevention, High risk places, Condom distribution, Condom availability, Condom use

## Abstract

**Background:**

A number of studies from countries with severe HIV epidemics have found gaps in condom availability, even in places where there is a substantial potential for HIV transmission. Although reported condom use has increased in many African countries, there are often big differences by socioeconomic background. The aim of this study was to assess equity aspects of condom availability and uptake in three African districts to evaluate whether condom programmes are given sufficient priority.

**Methods:**

Data on condom availability and use was examined in one district in Kenya, one in Tanzania and one in Zambia. The study was based on a triangulation of data collection methods in the three study districts: surveys in venues where people meet new sexual partners, population-based surveys and focus group discussions. The data was collected within an overall study on priority setting in health systems.

**Results:**

At the time of the survey, condoms were observed in less than half of the high risk venues in two of the three districts and in 60% in the third district. Rural respondents in the population-based surveys perceived condoms to be less available and tended to be less likely to report condom use than urban respondents. Although focus group participants reported that condoms were largely available in their district, they expressed concerns related to the accessibility of free condoms.

**Conclusion:**

As late as thirty years into the HIV epidemic there are still important gaps in the availability of condoms in places where people meet new sexual partners in these three African districts. Considering that previous studies have found that improved condom availability and accessibility in high risk places have a potential to increase condom use among people with multiple partners, the present study findings indicate that substantial further efforts should be made to secure that condoms are easily accessible in places where sexual relationships are initiated. Although condom distribution in drinking places has been pinpointed in the HIV/AIDS prevention strategies of all the three countries, its priority relative to other HIV/AIDS measures must be reassessed locally, nationally and regionally. In practical terms very clear supply chains of condoms to both formal and informal drinking places could make condom provision better and more reliable.

## Background

Since prehistoric times different types of penis covers have been used to provide protection during combat and to promote fertility. The invention of a sheath that fitted the penis and protected against syphilis was described as early as 1564 [1]. Condoms later turned out to be effective in preventing pregnancy [2,3], sexually transmitted infections in general, and have been found to reduce the risk of transmission of HIV during sexual intercourse by approximately 90% [4,5]. This makes the condom one of the most effective preventive measures that have been developed so far against HIV infection.

In communities with a high prevalence of HIV in the general population, everyone who engages in unprotected sex is theoretically at risk of infection. Actual high risk groups in such populations consequently do not only include sex workers and men who have sex with men. The need to reach people who are likely to take risks but who do not belong to traditional high risk groups has increased the interest in targeting HIV prevention at high risk places rather than specific population groups. During the last decade a number of studies have been conducted around the world on condom availability and HIV prevention in places where there is a high potential for HIV transmission, such as places where people meet new sexual partners. Many of these studies have found gaps that should be of great concern for HIV prevention programmes [6-14]. A systematic review of studies published between 1988 and September 2007 on structural-level interventions to improve condom availability or accessibility, identified one randomised controlled trial and 7 studies with repeated cross-sectional designs that had been conducted in bars, night clubs or brothels, and all of these found increased condom use with new or casual partners [15]. Among more recent intervention studies on the efficacy of condom distribution to venues where people meet sexual partners, one non-randomised study (with a control group) conducted in Zambia found increased condom use with new partners [16], whereas one randomized controlled cluster trial in Jamaica and one non-randomised study from gay bath-houses in Taiwan found non-significant differences in consistent condom use with casual partners. However, in the two latter studies there were substantial problems with the implementation of the intervention or loss to follow-up, respectively, and this may have reduced the apparent effectiveness of the interventions [17,18].

The national HIV/AIDS prevention strategies in Kenya, Tanzania and Zambia officially include condom distribution and condom promotion [19-22]. In all the three countries free condoms may be ordered from the national medical store department by the provincial/regional and district health teams, employing the same system that is used for procuring other medical supplies. From the district level, condoms are distributed to public health facilities. Private health facilities in Tanzania and Zambia may also receive free condoms upon request. Although bars, other drinking places and lodgings are specifically mentioned as important distribution points for condoms in the national HIV/AIDS strategies in the three countries [19-21], there are currently no guidelines for the condom supply chain to drinking places. Thus the presence of free condoms in such places depends on the efforts of the District Health Management Team (DHMT), the District AIDS coordinator or local NGOs. In addition to the distribution of free condoms, commercial and semi-commercial condoms are sold in pharmacies, shops and private health facilities, and sometimes in drinking venues.

Considering that the actual availability of condoms depends on the efforts of district health management teams and local NGOs, local availability is sensitive to district priorities and may thus deviate from what is stated as an objective in national strategy documents. The aim of this study was to assess equity aspects of condom availability and uptake in three African districts: Malindi in Kenya, Mbarali in Tanzania and Kapiri Mposhi in Zambia, to inform local and national policy-makers whether the condom programmes are given sufficient priority in these districts. The data utilized in this paper was collected in 2007 and 2008. At the time, condom distribution to formal and informal drinking venues was not budgeted for specifically by the three DHMTs. Instead it was supposed to be carried out by health workers as part of their duties in Mbarali and by NGOs providing socially marketed condoms in Kapiri Mposhi and Malindi. The objectives of this study were to assess whether the availability of condoms and HIV educational materials differed between different types of places where people meet new sexual partners; to examine urban/rural differences in perceptions of overall condom availability; and to assess reported condom use by socioeconomic profile in the three districts.

## Methods

### Study settings

The data for the study was collected as part of an EU-funded intervention study “REsponse to ACcountable priority setting for Trust in health systems” (REACT) in Malindi in Kenya, Mbarali in Tanzania and Kapiri Mposhi in Zambia. The aim of the intervention was to strengthen fair and participatory priority setting processes in the three district health management teams. The intervention targeted priority setting processes in general and did not focus on particular diseases or service domains. However, to assess the equity, quality of and trust in health services provided at baseline, four areas were selected: HIV/AIDS, malaria, emergency obstetric care, and generalized care. The methodology across the selected service areas at the baseline assessment included a population-based questionnaire survey, qualitative data gathering and specific studies of service areas [23]. The baseline data collection was carried out in 2007 and 2008. Prior to the baseline studies, consultations with the DHMTs were conducted to obtain permission to carry out the study, and the principles of the priority setting framework called Accountability for Reasonableness were explained. The application of the framework started at the end of 2008, after the baseline data collection had been completed. The districts were selected because they were assessed to be representative of district health systems within their country and to have similar disease burdens. The HIV prevalence estimates in the three selected districts were all higher than the overall national estimates in the three countries, and thus HIV/AIDS was an important service area. Malindi (Coast province) is a popular tourist destination. The district had an estimated population of 374,000 in 2008 with an HIV prevalence of 16% [24]. Mbarali (Mbeya region) is a primarily rural district. The estimated population of the district was 276,000 in 2008 [25] and the HIV prevalence in Mbeya region was 7.9% [26]. Kapiri Mposhi (Central province) is also a primarily rural district, but has an urban centre that is situated along the main road and the railway line to Tanzania. The estimated population of this district was 289,000 in 2007 [27]. No overall HIV prevalence estimate for the district is available, but a population-based survey in 2003 estimated it to be 13% in rural areas [28] while the prevalence among pregnant urban women was 25% in 2008 (according to unpublished ANC-data).

### Data collection in places where people meet new sexual partners

Data on HIV prevention and condom distribution in places with a big potential for HIV transmission was collected using a short version (including the first two of the three standard phases) of the Priorities for Local AIDS Control Efforts (PLACE) method. The PLACE method was developed to rapidly assess the presence and need for HIV preventive materials and activities in places where there is a high risk of HIV transmission. In settings with a severe heterosexual HIV epidemic, venues where people meet new sexual partners and casual sex may take place are assumed to be places where the risk of acquiring the infection is high [10]. In the first phase of the PLACE studies, local people (sampled by convenience in urban and peri-urban areas of the districts) were asked to name all the venues in the district where men or women met new sexual partners or were paid to have sex. The interviewers continued interviewing new people until no new places were mentioned. In Malindi and Mbarali, people encountered in the streets, in shops, bars and health facilities were interviewed, whereas in Kapiri Mposhi, people working and socializing in bars, nightclubs and hotels were approached and asked to name relevant venues. In the second study phase, the interviewers sought all the mentioned venues. In the venues that were located, one person (a member of staff or a patron, referred to as “venue representative”) was asked whether people met sexual partners there, whether condoms were available and whether other HIV preventive activities ever had been organized there. The interviewers also observed whether HIV-related educational materials, including condoms, were present during the visit. (The third phase of the PLACE-method, in which individuals socializing in venues where people meet new sexual partners are interviewed about partnership formation and sexual behaviour, was not included in the present study due to lack of resources.) The data collection was conducted in 2007 in Kapiri Mposhi and in 2008 in Malindi and Mbarali districts.

### Data on condom use, sexual behaviour and perceptions of condom availability in the general population

In all the three districts a population-based survey based on three-stage random cluster sampling was conducted in 2007.The target sample size was 2000 in each district. Rural and urban clusters (corresponding to census enumeration areas) in the districts were stratified and selected with probability proportional to size. In Malindi 10 urban clusters and 16 rural clusters were selected, whereas in Mbarali and Kapiri Mposhi the corresponding numbers were 25 rural clusters and 20 urban clusters. Within each cluster, a complete listing of all households was carried out. In each urban cluster 25 households were selected, whereas 20 households were selected in each rural cluster. Within the selected households one woman and one man aged between 15 and 49 were randomly selected and asked for an interview. In case of refusal, another household was randomly selected from the list in the respective enumeration areas. The questionnaires included sections on socio-demographic characteristics and sexual behaviour. The overall response rate was 93% and did not differ substantially by district.

### Data entry and statistical analysis

The data was double-entered in Epiinfo. The data from high risk venues (PLACE-data) was analysed using SPSS version 15. We compared the types of venues identified as places where people met sexual partners, the reported frequency of different activities in the visited venues, and the reported and observed presence of prevention materials between the three districts. Differences between subgroups were tested using the Pearson’s Chi square test or the contingency coefficient (the latter was used when >20% of the cells had counts less than 5 or any cells had counts <1) in Tables 1 and 2, or Chi square test for linear trend (in Table 3). We used logistic regression to assess whether differences in observed condom availability between different types of sites and between the three districts were significant. “Condoms observed by the interviewer” (yes or no) was used as the outcome and type of site or district, respectively, were used as covariates. We also assessed whether the observed differences in condom availability between the districts could be due to differences in venue types by including both the covariates at the same time.

**Table 1:** Characteristics of places where people meet new sexual partners, Phase 2 of PLACE studies

		**Malindi**		**Mbarali**		**Kapiri Mposhi**		
		**%**	***N***	**%**	***N***	**%**	***N***	**p**
Place verification	Venue found	86	*117*	98	*48*	98	*43*	**0.003**
	Venue found, but no willing respondent found	0		2		0		
	Venue not found	15		0		0		
	Venue closed temporarily	0		0		2		
	Venue closed permanently	1		0		0		
Type of place	Informal drinking place	49	*97*	25	*48*	0	*41*	**<0.001**
	Bar/restaurant	30		27		90		
	Night club	7		0		5		
	Hotel/guest house	8		12		5		
	Local brew places	0		17		0		
	Rice mill	0		8		0		
	Bus station	0		4		0		
	Market	3		6		0		
	Park/beach	1		0		0		
	Church	1		0		0		
	Other	1		0		0		
Position of respondent	Staff	87	*98*	73	*48*	43	*42*	**<0.001**
	Patron	13		27		57		
Men meet new female sexual partners here		82	*97*	92	*47*	88	*42*	0.517
Women meet new male sexual partners here		82	*97*	83	*47*	88	*42*	0.802
Men meet new male sexual partners here		14	*93*	2	*47*	2	*42*	**0.011**
Women meet new female sexual partners here		-	*-*	2	*47*	-	*-*	
Women come to sell sex		38	*97*	70	*47*	86	*42*	**<0.001**
Men come to sell sex		18	*97*	28	*47*	64	*42*	**<0.001**
Ever HIV prevention activities here		53	*98*	83	*47*	69	*42*	**0.002**
HIV-related lectures/seminars		8	*98*	49	*47*	29	*42*	**<0.001**
HIV-related pamphlets/leaflets		5	*98*	22	*47*	12	*42*	**0.005**
HIV-related posters		10	*98*	17	*47*	45	*42*	**<0.001**
Condom distribution		51	*98*	85	*47*	79	*42*	**<0.001**
HIV-related peer education		0	*98*	26	*47*	17	*42*	**<0.001**
How often condoms available	Always	29	*97*	67	*40*	51	*37*	**<0.001**
	Sometimes	23		23		38		
	Never	48		10		11		
Condoms at time of visit (according to venue representative)		33	*97*	57	*47*	57	*42*	**0.012**
Willing sell/distribute condoms (if respondent was staff)		84	*83*	80	*35*	94	*16*	0.456
Posters observed by interviewer		7	*99*	12	*48*	33	*42*	**<0.001**
Pamphlets observed by interviewer		1	*99*	23	*48*	0	*42*	**<0.001**
Condoms observed by interviewer		31	*99*	44	*48*	57	*42*	**0.014**

**Table 2:** Sociodemographic characteristics of respondents, population-based surveys

		**Malindi**					**Mbarali**					**Kapiri Mposhi**				
		**Rural**		**Urban**		**Pearson chi square**	**Rural**		**Urban**		**Pearson chi square**	**Rural**		**Urban**		**Pearson chi square**
		**%**	**n**	**%**	**n**	**P**	**%**	**n**	**%**	**n**	**p**	**%**	**n**	**%**	**n**	**p**
Gender	Male	50	1216	50	628	0.481	49	1057	50	935	0.189	45	931	43	919	0.214
	Female	50		50			51		50			55		57		
Age	15-19	15	1206	11	627	**<0.001**	8	1057	12	935	**0.046**	12	931	12	911	0.089
	20-24	16		24			18		19			15		18		
	25-29	17		21			21		20			20		20		
	30-39	31		30			36		32			32		33		
	40-49	21		14			17		17			21		16		
Marital status	Single, never married	21	1214	29	627	**0.005**	10	1057	17	935	**0.019**	15	931	21	916	**0.015**
	Married or cohabiting	74		63			90		83			76		71		
	Widowed, divorced or separated	5		8			0		0			10		9		
Educational level	Low	40	1203	30	625	**0.021**	22	1057	25	935	0.123	40	931	34	911	0.124
	Medium	34		35			71		65			34		32		
	High	25		35			7		10			26		34		

**Table 3:** Relationship between claimed frequency of condom availability in venue and condom presence at the time of visit

		**Malindi**			**Mbarali**			**Kapiri Mposhi**		
		**%**	***N***	**P for linear trend**	**%**	***N***	**P for linear trend**	**%**	***N***	**P for linear trend**
Condoms observed by interviewer	Condoms always available	96	*28*	**<0.001**	59	*27*	**0.001**	84	*19*	**<0.001**
	Condoms sometimes available	18	*22*		22	*9*		50	*14*	
	Condoms never available	0	*47*		0	*4*		0	*4*	

The population-based data was analysed with Stata Intercooled 10, adjusting for the effects of cluster sampling. The latter analyses were stratified by country and urban/rural residence. We compared the percentages reporting two or more partners in the previous 12 months, condom use during the previous sexual intercourse, condom use with the previous casual partner and always condom use with casual partners (the denominator for the latter two were those reporting casual partners in the previous 12 months) by two proxies of relative socioeconomic status, i.e. education and wealth, because of a particular interest in equity. Separate wealth indices were created for urban and rural areas of each district. They were based on household ownership of assets and food availability in the previous year. We used principal components analysis to assign the indicator weights (with the SPSS factor analysis procedure), and the first of the factors obtained was used. We then created wealth tertiles of similar sample sizes. Educational tertiles were created separately for urban and rural men and women in each country, aiming for categories of similar sample size (see Additional file 1: Table S1 for cut-off points for the educational tertiles). Although the absolute level of reported multiple partnerships and condom use was systematically higher among men than women, we also pooled men and women in the analyses which assessed differences by education to gain power since the effect of education was similar for men and women from the same area. Analyses stratified by sex are described in the text but not shown in the tables. Differences between subgroups in relation to reported number of sexual partners and condom use were tested using log-binomial regression based on the generalized linear model (in Table 4). Multinomial logistic regression was used to compare urban and rural respondents’ perceptions of condom availability within their district (in Table 5).

**Table 4:** Reported sexual behaviour by respondents, population-based surveys

**Variable**	**Educational level**	**Rural Malindi**				**Urban Malindi**				**Rural Mbarali**				**Urban Mbarali**				**Rural Kapiri Mposhi**				**Urban Kapiri Mposhi**			
		**%**	***n***	**aRR**	**95% CI**	**%**	***n***	**aRR**	**95% CI**	**%**	***n***	**aRR**	**95% CI**	**%**	***n***	**aRR**	**95% CI**	**%**	***n***	**aRR**	**95% CI**	**%**	***n***	**aRR**	**95% CI**
Had two or more sexual partners in previous 12 months	Low	19	*460*	Ref.		17	*170*	Ref.		9	*209*	Ref.		8	*227*	Ref.	9	10	*373*	Ref.		15	*303*	Ref.	
	Middle	20	*400*	1.10	0.75-1.62	21	*211*	1.20	0.87-1.64	12	*710*	**1.32**	**1.02-1.72**	12	*602*	**1.52**	**1.05-2.21**	14	*316*	1.40	0.83-2.35	11	*291*	0.76	0.46-1.25
	High	14	*287*	0.83	0.64-1.07	12	*210*	0.72	0.40-1.29	20	*74*	**2.15**	**1.27-3.66**	13	*94*	1.75	0.91-3.38	15	*236*	**1.51**	**1.03-2.23**	8	*303*	**0.56**	**0.33-0.95**
	*Total*	*18*	*1147*			*17*	*591*			*12*	*993*			*11*	*923*			*13*	*925*			*11*	*897*		
Condom use at previous sexual intercourse	Low	9	*447*	Ref.		24	*171*	Ref.		22	*184*	Ref.		17	*199*	Ref.		7	*348*	Ref.		17	*264*	Ref.	
	Middle	19	*368*	**1.78**	**1.20-2.65**	30	*199*	1.22	0.99-1.50	25	*633*	1.14	0.84-1.55	27	*561*	**1.59**	**1.09-2.33**	9	*292*	1.27	0.88-1.84	13	*255*	0.76	0.47-1.22
	High	22	*246*	**2.15**	**1.41-3.25**	28	*193*	**1.31**	**1.02-1.67**	30	*66*	1.37	0.79-2.39	24	*76*	1.40	0.83-2.35	12	*210*	**1.79**	**1.08-2.97**	21	*270*	1.21	0.82-1.78
	*Total*	15	*1061*			27	*563*			25	*883*			24	*836*			9	*850*			17	*789*		
Condom use at previous sexual intercourse with casual partners	Low	41	*61*	Ref.		73	*26*	Ref.		88	*8*	Ref.		60	*10*	Ref.		31	*48*	Ref.		55	*53*	Ref.	
	Middle	65	*54*	**1.43**	**1.05-1.94**	68	*40*	0.93	0.74-1.17	80	*44*	**0.83**	**0.72-0.95**	74	*39*	1.49	0.63-3.50	36	*47*	1.32	0.83-2.10	56	*39*	1.06	0.75-1.51
	High	61	*28*	1.36	0.90-2.03	87	*30*	1.18	0.96-1.45	67	*9*	**0.60**	**0.37-0.98**	100	*5*	2.14	0.98-4.66	43	*37*	1.39	0.96-2.01	73	*37*	**1.41**	**1.10-1.82**
	*Total*	54	*143*			75	*96*			79	*61*			74	*54*			36	*132*			60	*129*		
Always use condom with casual partners	Low	39	*62*	Ref.		62	*26*	Ref.		75	*8*	Ref.		50	*10*	Ref.		17	*48*	Ref.		34	*53*	Ref.	
	Middle	50	*54*	**1.24**	**1.00-1.53**	58	*40*	0.95	0.63-1.43	68	*44*	**0.84**	**0.75-0.94**	54	*39*	0.88	0.37-2.12	18	*45*	1.10	0.41-2.90	41	*39*	1.25	0.76-2.05
	High	54	*28*	1.26	0.83-1.90	73	*30*	1.24	0.79-1.94	67	*9*	0.66	0.40-1.09	80	*5*	1.51	0.72-3.19	22	*37*	1.23	0.59-2.58	35	*37*	1.09	0.66-1.82
	*Total*	*46*	*144*			*64*	*96*			*69*	*61*			*56*	*54*			*18*	*130*			*36*	*129*		

**Table 5:** Perceived availability of condoms in own district, population-based surveys

	**Malindi**						**Mbarali**						**Kapiri Mposhi**					
	**Rural**		**Urban**				**Rural**		**Urban**				**Rural**		**Urban**			
	**%**	**n**	**%**	**n**	**OR**	**95% CI**	**%**	**n**	**%**	**n**	**OR**	**95% CI**	**%**	**n**	**%**	**n**	**OR**	**95% CI**
Never	12	881	13	507	0.98	0.42-2.31	15	824	3	719	0.71	0.27-1.82	6	802	3	733	**0.36**	**0.17-0.80**
Rarely/sometimes	18		4		**0.17**	**0.07-0.41**	26		28		0.25	0.06-1.05	31		10		**0.23**	**0.15-0.36**
Mostly/always	71		83		Ref.		59		69		Ref.		64		87		Ref.	

### Focus group discussions

Eight focus group discussions (FGDs) were conducted in each study district (four with urban participants and four with rural participants) with the following groups of outpatients: females, males, pregnant women, and adolescents aged 18 to 24 years. Each group consisted of 6–12 participants. Recruitment of participants was done at the health facilities that served the clusters sampled for the population-based surveys in order to enrol individuals who had recent experiences with health services and thus would be able to comment on the perceived equity and quality of the services provided and their own trust in these services. However, the venue for discussion was outside the health facilities to promote open expression of opinions. The discussions were based on a semi-structured discussion guide that included topics related to generalized care, malaria, and HIV. We analysed the discussion of condom availability and costs. The discussions in Kapiri Mposhi were conducted in Bemba, whereas Swahili was used in Mbarali and Malindi. The discussions were tape-recorded in all three districts. In Kenya and Tanzania the discussions were transcribed verbatim and later translated into English, whereas the translation was done during the transcription process in Zambia. During the translation process substantial emphasis was placed on retaining culturally embedded expressions.

The qualitative analysis was guided by the ‘framework approach’ [29], and implied reading carefully through all the transcripts. The detailed coding was done with a focus on the sections relating to perceived condom availability/accessibility and perceived high risk places, which were the predetermined sub-themes. The codes were during the next phase merged into larger categories or sub themes with similar content. Continuous comparisons were done both within and between the transcripts, and we searched for both commonalities and differences within the emerging topics to explore the range of views on the selected subthemes in each district.

### Ethical considerations

Ethical approvals for the REACT study were obtained from the Kenya Medical Research Institute (KEMRI) and the National Ethical Review Committee (NERC) of Kenya, the Medical Research Coordinating Committee (MRCC) of the National Institute for Medical Research (NIMR) in Tanzania, and the University of Zambia Research Ethics Committee. Participation in the PLACE-surveys and the focus groups were based on oral informed consent, whereas participation in the population-based surveys was based on written consent. In cases where the selected household member was aged between 15 and 18 years, permission was also sought from a guardian and the adolescent was asked to assent. The participants were informed that the information they provided would be kept confidential and anonymous.

## Results

### Places where people meet new sexual partners and availability of condoms and educational materials

During the first phase of the PLACE-study, 28 persons in Malindi (among whom one refused), 120 in Kapiri Mposhi and 36 in Mbarali (among whom one person did not contribute because he was not from the area) were asked to name places where people met new sexual partners. The people who were approached were youths, shop staff, community leaders, bar workers and health personnel. In Malindi 117 venues were named, whereas 48 and 43 places were named in Mbarali and Kapiri Mposhi, respectively. Most of the venues in Malindi and Mbarali were informal drinking places (serving alcohol without a license), bars or restaurants. In Kapiri Mposhi, almost all the mentioned venues were bars or restaurants. During phase two, more than 80% of venue representatives interviewed agreed that men and women came to meet new sexual partners of the opposite sex in their venue, and the majority in Mbarali and Kapiri Mposhi indicated that women came to sell sex. However, only 40% of venues in Malindi were reported to be places where women sold sex (Table 1).

There was a considerable difference in the percentage of venues where HIV prevention activities were said to have ever taken place, from half of the venues in Malindi to 83% in Mbarali. Condom distribution was the most common HIV preventive activity reported in all three districts. However, in contrast to Mbarali where it was reported that condoms were always available in two thirds of venues, only half of the venues in Kapiri Mposhi and one third of venues in Malindi reported the same (Table 1). As many as 70% of informal drinking venues in Malindi reported to never have condoms available. Condoms were more likely to be observed in bars/restaurants (OR 9.9, 95% CI 4.1-23.6), night clubs (OR 12.7; 95% CI 2.6-61.5) and hotels (OR 8.2, 95% CI 2.4-28.3) than in informal drinking places. Based on the interviewers’ observations, condoms appeared to be more often available in venues in Kapiri Mposhi than in Malindi (OR 2.92, 95% CI 1.39-6.16). However, when we adjusted for types of sites identified in the districts, this difference was no longer significant (adjusted OR 0.90, 95% CI 0.37-2.17). In cases where the venue representatives claimed that condoms were present at the time of the visit, this was confirmed by the interviewers’ own observations in almost all the cases in Malindi (31 out of 32) and Kapiri Mposhi (21 of 21), but only in 21 out of 27 cases in Mbarali. Venues which were reported to always have condoms available were more likely to have them at the time of the interviewers’ visit. However, only 59% of the venues in Mbarali where condoms were claimed to always be present, had condoms at the time of the interviewers’ visit (Table 3). In all three districts, HIV-related posters were observed by the interviewers in two thirds of the venues where the venue representative reported that posters had been present at some point (Table 1).

### Socioeconomic differences in condom use, sexual behaviour and urban–rural differences in perceptions of condom availability

In the population-based surveys, the majority of the respondents were married (Table 2). Men were more likely than women to report multiple partners and condom use in both rural and urban areas of all the three districts. Overall reported condom use with the last casual partner was more than 50% in all districts, except in rural Kapiri Mposhi where it was reported by one third. The highest reported rate of condom use with casual partners was in Mbarali, where this was reported by approximately 3 out of 4 in both rural and urban clusters (Table 4). Educated men and women in urban and rural Malindi and urban Kapiri Mposhi were less likely to report two or more partners in the previous year (but the difference was only significant for urban women in Kapiri Mposhi). The opposite tendency was observed for rural men in Kapiri Mposhi and Mbarali, urban and rural women in Mbarali (pooled results shown in Table 4). Respondents with little education tended to be less likely than the more educated to report condom use with casual partners (except in rural Mbarali where the most educated were less likely to report condom use: RR 0.60; 95% CI 0.37-0.98) (Table 4). There were no systematic differences in condom use with casual partners by wealth (results not shown). In Malindi and Kapiri Mposhi reported condom use was higher among urban than rural respondents. However, in Mbarali the opposite was the case (but the difference was only significant for “always use condom with casual partners”) (Table 4). Rural respondents were more likely than urban respondents to report that condoms were rarely/sometimes or never available in their district (Table 5).

### Findings from focus group discussions

Bars and hotels were spontaneously mentioned by focus group participants as places where HIV transmission could take place. People socializing in these places were perceived to take risks:

“… AIDS is mostly found in the bar, because when one gets drunk they usually have the perception … you can get a lady and sleep with her.)… This time these girls look fat and when you look at them you wouldn’t even suspect (HIV)…”

Participant 4, FGD with males aged 15–34 years, Kapiri Mposhi.

When asked a general question about condom availability in their district, the participants in the focus groups claimed that male condoms were readily available from shops (where condoms could be bought), and from health facilities (where whole boxes of free condoms could be obtained). In the evenings free condoms were said to be more difficult to find because service hours at the clinics ended at 16:00, (but participants in Mbarali mentioned that condoms were sold at night when there was a festival). Participants mentioned that condoms were not always available in rural areas. Although the majority of the participants found condoms to be affordable, others expressed that condoms should be cheaper, and they missed information about where free condoms could be obtained:

“P3: … Maybe there are those that some say are provided for free at the district hospital. .. These things are not open and they aren’t widely known in the target community. …”

FGD with urban men, Mbarali.

## Discussion

Data from the three study districts in Kenya, Tanzania and Zambia suggests important gaps in the availability of condoms in places where people meet new sexual partners. Despite bars and lodgings being mentioned as important distribution points for condoms in the national condom strategies of the three countries [19-21] and the districts on paper having local strategies to distribute condoms to such places, condoms were only observed by the interviewers in a third of venues in Malindi, in less than half in Mbarali and just over half the venues in Kapiri Mposhi. We found that informal drinking places were least likely to have condoms available. Since half the identified venues in Malindi were such informal drinking places, this explained why the overall proportion of venues where condoms were reported to be available was particularly low there. Condoms were perceived to be less available in rural than in urban areas by participants in the population-based surveys and the FGDs, and there was a tendency of rural respondents (in Malindi and Kapiri Mposhi) and less educated people (in Malindi, Kapiri Mposhi and urban Mbarali) to be less likely to report condom use with casual partners. This indicates that further efforts should be made to improve condom availability and promote condom use among these groups.

In many societies around the world, having multiple sexual partners has traditionally been associated with higher socioeconomic status. In times of HIV, sexual norms have changed and people with formal education have in many countries adapted more rapidly to the messages of HIV prevention campaigns than less educated people because they tend to have the knowledge, self-efficacy and self-confidence to achieve behaviour change [30-32]. The findings from the population-based surveys which revealed that more educated respondents were less likely to report multiple partners in Malindi and urban Kapiri Mposhi and tended to report more condom use with casual partners in all three districts (except in rural Mbarali) are in line with this pattern. However, educated respondents in urban and rural Mbarali and in rural Kapiri Mposhi were, somewhat surprisingly, more likely to have multiple partners. These findings may indicate that the expected process of change in sexual norms and behaviour related to education has taken more time in Mbarali and rural Kapiri Mposhi. In Kapiri Mposhi (and in Malindi where rural respondents overall were less likely to report condom use) HIV prevention campaigns may not have reached rural areas with the same intensity as urban areas. Since improved condom availability has been found to result in higher levels of condom use [15,16], it is reasonable to believe that improved availability in rural areas would contribute to smaller differences between urban and rural residents in Malindi and Kapiri Mposhi in condom use with casual partners. In Mbarali, the small difference between clusters coded as urban and rural in the reporting of sexual behaviours and condom availability probably reflected that clusters classified as urban were actually periurban and not very different from the rural clusters. However, it is difficult to explain why condom use with casual partners was more common in rural than periurban clusters. Anyhow, the reported condom use with the previous casual partner in the three districts was higher (except in rural Kapiri Mposhi) than the level reported with non-marital, non-cohabiting partners by men and women in national population-based surveys conducted between 2007 and 2009 in the respective countries [33-35].

The data triangulation (including two survey types, observations and FGDs) allowed us to validate information from one data source against another. We found that the perceptions of condom availability reported in the population-based surveys were in line with the perceptions expressed by focus group participants, i.e. the majority expressed that condoms were available in their district. However, the questions posed in both the population-based surveys and the FGDs were unspecific, and specific questions about condom availability at night or in places where people meet new sexual partners were not posed. This made a comparison with the condom availability data from places where people meet new sexual partners difficult. It is likely that the participants in the population-based surveys and focus group discussions referred to daytime availability from health clinics and shops when they reported that condoms were available since these were the main sources mentioned by the focus group participants. The relatively high proportions reporting condom use in the population-based surveys (except in rural Kapiri Mposhi) suggest that many people in these three districts did obtain condoms from clinics or shops. However, based on our findings from venues where people met new sexual partners, it seems likely that the respondents would have answered differently if they had been specifically asked about condom availability in drinking places at night.

National surveys conducted by Population Services International (PSI) in Tanzania and Zambia between 2006 and 2009 have also found shortcomings in the distribution of condoms and condom promotional materials in high risk zones (e.g. lack of coverage or stock-outs), particularly in rural areas [36-39]. Our findings from Mbarali, Kapiri Mposhi and Malindi indicate that there is a need for a better system for monitoring the condom availability in high risk places. In all the three districts, drinking establishments sometimes requested condom replenishments, but this depended entirely on the initiative of the individual venue owners. In Mbarali there were no specific guidelines concerning how often health workers should supply condoms, whereas in Kapiri Mposhi, NGOs were supposed to make weekly visits to high risk places. Unfortunately we do not have information about how often NGOs made such visits in Malindi in 2008. Anyhow, the data presented in this study indicates that more frequent visits or closer collaboration with venue owners than what was in place in 2007/2008 is needed in all three districts to ensure good availability of condoms. Closer collaboration with the drinking venues seems possible since the majority of staff interviewed in the venues during the second phase of the PLACE-studies expressed willingness to distribute or sell condoms.

We found that informal drinking places in Malindi rarely had condoms available. This may be because informal drinking places are less well established than drinking places which have a license to serve alcohol, and they may thus have been more likely to be missed by NGOs supplying socially marketed condoms. Specific efforts seem to be needed to secure that these venues are mapped and included as distribution points for condoms. Although we have not identified any studies that have compared the effectiveness of condom provision in high risk places versus health clinics, we believe that condom availability in places where people decide to have sex, including informal drinking places, is of vital importance because sex is often not planned. With easy access at that moment, less effort and personal motivation would be demanded to obtain them.

Some FGD participants expressed concerns about limited access to free condoms, whereas others found the ones available from shops to be affordable. The Kenyan “National Condom Policy and Strategy 2001-2005” states that “In order to improve the efficiency of condom distribution and reduce waste, health providers will be encouraged to charge clients who are able to contribute toward their own protection.” (p. 14) [19]. However, introducing payments on services that have previously been free is risky, especially in resource-constrained settings. Increased prices of condoms in the US, Zimbabwe and Bangladesh in the 1990s were documented to reduce demand, and in Bangladesh reducing the prices again lead to sales increasing to previous levels [40-42]. The Kenyan government has tried to avoid such scenarios by providing free condoms from public facilities to people who cannot afford to buy them [19], and available data indicates that a drop in condom demand has not occurred [33]. However, based on findings from the US it is possible that it would be more effective and less administratively demanding to provide drinking venues with free condoms than to offer subsidized ones [42].

Some of the information provided by venue representatives in places where people met new sexual partners could be validated against the observations of the interviewers, and we found that the observations normally confirmed what was reported. For example, condoms were more likely to be observed in venues where they were claimed to always be available than in venues where the representative stated that they were less frequently present. However, we cannot rule out a certain level of report bias, since we found that in Mbarali 40% of the venues where condoms were claimed to be ‘always available’ did not have condoms when the interviewers checked. Venue representatives may have been tempted to over-report HIV preventive activities taking place in their venue since it is likely that they perceived this as socially desirable. There may also have been a selection bias in the PLACE-study in Kapiri Mposhi since a comparison with a similar survey conducted two years earlier in the same district revealed that the interviewers did not obtain the names of the full range of existing venues (125 places were named in 2005, of which 101 were found). More than 30 of the venues in the 2005-survey were of types not mentioned at all during our listing in 2007, e.g. informal drinking places, bus stations, markets, shops, truck stops, fields and sports events [6]. The discrepancy may be related to fewer people being interviewed in the first phase of our survey or the interviewers may have posed the question in a way that limited the variety in responses. In the two other districts it is difficult to judge how many relevant venues we failed to list. Although sampling of individuals in the first phase of the PLACE-study only ended when new venue names stopped coming up, it is possible that more venues would have been mentioned if a higher number of people had been interviewed. We nonetheless believe that we obtained a more representative sample of venues in Malindi and Mbarali as there was greater variation in the types of places listed.

## Conclusion

The data in this study indicate important gaps in the availability of condoms in places where people meet new sexual partners in the three study districts, particularly in informal drinking places. From an equity perspective it is important that efforts to provide condoms in high risk places target the whole range of venue types where people meet new sexual partners. Although condom use with non-marital, non-cohabiting partners has increased in the three countries [33-35,43] and the HIV incidence is estimated to have declined by more than 25% in Tanzania and Zambia after 2000 [44], efforts to bring the HIV incidence further down are nevertheless highly required. Considering the resources that are invested in the provision of HIV treatment and in research on new biomedical preventive technologies [45], it is striking that three decades into the epidemic, it is still difficult to raise enough resources to make condoms free and easily accessible where they are needed. Condoms provide much higher protection than what the microbicides [46] and vaccines [47] tested so far seem to be able to offer. According to the Accountability for Reasonableness-framework, which was tested as a priority setting tool at district level by the REACT project [23], there is a need to build consensus among local stakeholders about how equitable and easy access to condoms may be achieved. Priority setting related to HIV prevention should involve those at risk or already infected, owners of venues where new sexual partners meet, health teams and other immediate providers. We would recommend that higher priority is given to condom distribution in rural areas in general and to drinking places and lodgings in particular. It is also vital that informal drinking places are not missed in such efforts. Establishing formal guidelines for condom supply chains, including the involvement of venue owners, may be helpful at district level to ensure reliable provision and frequent replenishing of free or inexpensive condoms in venues where people meet new sexual partners.

## Competing interests

The authors declare that they have no competing interest.

## Authors’ contributions

IFS designed the questionnaires for the PLACE-studies and was active in the design of the population-based surveys and the interview guide for the focus group discussions (FGDs), analysed the data, interpreted the findings and drafted the manuscript. AB interpreted the findings and revised the manuscript. EHS conducted some of the FGDs in Mbarali, interpreted the findings and revised the manuscript. EM collected the PLACE-data in Mbarali, interpreted the findings and revised the manuscript. CM was active in the design of the population-based surveys, coordinated the data collection in Kapiri Mposhi, interpreted the findings and revised the manuscript. JZ interpreted the findings and revised the manuscript. JB sent the application for funding to the EU and coordinated the project, its design, data collection, interpreted the findings and revised the manuscript. All authors approved the final version of the manuscript.

## Pre-publication history

The pre-publication history for this paper can be accessed here:

http://www.biomedcentral.com/1471-2458/12/1030/prepub

## Supplementary Material

Additional file 1 **Table S1.** Categorisation of educational tertiles based on years attended school for respondents aged 15–49 years. Click here for file
