# Supplementary material for: Condom availability in high risk places and condom use: a study at district level in Kenya, Tanzania and Zambia
Source: BMC Public Health. 2012 Nov 26;12:1030. doi: 10.1186/1471-2458-12-1030 (PMC3533956; doi:10.1186/1471-2458-12-1030)
Supplement: Additional file 1 — Table S1. Categorisation of educational tertiles based on years attended school for respondents aged 15–49 years. [file 1471-2458-12-1030-S1.doc]

**Supplementary table S1: Categorisation of educational tertiles based on years attended school for respondents aged 15-49 years**

|  | **Educational level** | **Malindi** | **Mbarali** | **Kapiri Mposhi** |
| --- | --- | --- | --- | --- |
| Rural men | Low | 0-5 | 0-6 | 0-6 |
|  | Middle | 6-8 | 7 | 7-8 |
|  | High | ≥9 | ≥8 | ≥9 |
|  |  |  |  |  |
| Rural women | Low | 0 | 0-6 | 0-5 |
|  | Middle | 1-6 | 7 | 6-7 |
|  | High | ≥7 | ≥8 | ≥8 |
|  |  |  |  |  |
| Urban men | Low | 0-7 | 0-6 | 0-7 |
|  | Middle | 8-11 | 7 | 8-10 |
|  | High | ≥12 | ≥8 | ≥11 |
|  |  |  |  |  |
| Urban women | Low | 0-5 | 0-6 | 0-6 |
|  | Middle | 6-9 | 7 | 7-8 |
|  | High | ≥10 | ≥8 | ≥9 |
